# Effect of electric and magnetic fields on impurity binding energy in zinc-blend symmetric InGaN/GaN multiple quantum dots

**DOI:** 10.1186/s40580-014-0025-3

**Published:** 2014-09-03

**Authors:** Ebrahim Sadeghi, Elham Naghdi

**Affiliations:** Department of Physics, Yasouj University, Yasouj, 75914-353 Iran

**Keywords:** Bessel function, Binding energy, External field, Multiple quantum dot, Whittaker function

## Abstract

The binding energy of ground state for hydrogenic impurity in multiple quantum dots is calculated in the framework of effective-mass approximation and using a variational method. It is shown that the binding energy is a function of the size of dots, impurity position and external fields strength. The binding energy has a maximum value when the impurity is located on the center of dots and decreases for other impurity positions. The external electric and magnetic fields change the magnitude and the position of peaks.

**PACS Codes** 73.20.D; 71.21.La; 71.55.Eq

## Background

The study of confined quantum systems has been the interesting subject of investigation since the beginning of quantum theory. The interest in the study of the physical properties of confined quantum systems such as quantum wells, wires, and dots, has increased, with the recent progress in semiconductor nanotechnology [1–5].

In recent years, theoretical and experimental investigations have been performed into the issue of the hydrogenic binding of an electron to a donor impurity which is confined within low-dimensional heterostructures [6,7]. The understanding of the electronic and optical properties of impurities in such systems is important because the optical and transport properties of devices made from these materials are strongly affected by the presence of shallow impurities.

The wide-band gap GaN material systems have attracted much attention for their applications in optoelectronic devices [8]. Built-in electric field is absent in zinc-blend (ZB) GaN structures because the spontaneous polarization does not exist in the ZB GaN due to the higher crystal symmetry. There has been a lot of work devoted to understanding of hydrogenic impurity states in ZB GaN quantum dots and quantum wire [9–14]. In all of those calculations, they are all base on single low-dimensional quantum structures. The impurity effects in ZB GaN-based multiple QDs have also been investigated theoretically [15,16]. In theses systems, wave function would penetrate more to the adjacent quantum dots if the barrier height or the barrier thickness is reduced.

On the other hand, the application of an external electric field can provide much valuable information about the confined impurities. Recent theoretical investigations predicated both the field induced level shifts and the field dependence of the carrier lifetime. Therefore, the impurity and the applied electric field effects on the optical properties of QDs are of great interest for fundamental physics and device applications [17,18].

To our knowledge, there have not been theoretical investigations on impurity states in ZB symmetric multiple GaN QDs under external electric and magnetic fields. In this paper the variational method is used for calculating the impurity binding energy in symmetric *I* *n* *G* *a* *N*/*G* *a* *N* multiple quantum dots. In this regard, a trial wave function based on the carrier wave function in cylindrical quantum dot is introduced and the energy is calculated. In Section 2, the Hamiltonian and the calculation method are given. The numerical calculations and discussion on typical InGaN/GaN material are presented in Section 3.

## Methods

**Figure 1:**
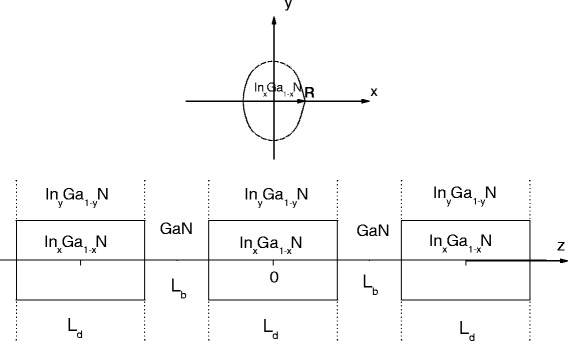
**Typical cross section of symmetric InGaN/GaN multiple quantum dots.**

We consider a cylindrical ZB symmetric *I* *n*_*x*_ *G* *a*_1−*x*_ *N* multiple QD of radius *R* and length *L*_*d*_ surrounded by two large energy gap materials *I* *n*_*y*_ *G* *a*_1−*y*_ *N* in the radial direction and GaN in the z-direction, Figure 1. Within the framework of effective mass approximation, the Hamiltonian description of an electron in the presence of an external field and hydrogenic impurity is given as

(1) $$ \hat{H}=\hat{H}_{0} -\frac{e^{2}}{\varepsilon |r-r_{i}|}  $$

with

*a) Electric field*

(2) $$ \hat{H}_{0}=\frac{\hat{P}^{2}}{2m^{*}} + V(\rho,z)+|e|Fz  $$

where *m* ^∗^, *F* and *V*(*ρ*,*z*) are the effective mass of charge carriers, electric field strength and the confinement potential. The confining potential is

(3) $${} V(\rho,z)= \left\{\! \begin{array}{ll} V(\rho) &\quad\!\! {\mathrm{z}_{1}<\mathrm{z}<\mathrm{z}_{2},\ \mathrm{z}_{3}<\mathrm{z}<\mathrm{z}_{4},\ \mathrm{z}_{5}<\mathrm{z}<\mathrm{z}_{6} } \\ V_{II} &\quad\!\! \text{otherwise}\;\; \end{array} \right.  $$

where

(4) $$ V(\rho)= \left\{ \begin{array}{ll} 0 & \quad{\rho\leq \mathrm{R} }\\ V_{I} & \quad{\rho >\mathrm{R} }\;\; \end{array} \right.  $$

Using the separation of variable, the eigenfunction of $\hat {H}_{0}$ is written as

(5) $$ \psi(\rho,\phi,z)=f(\rho) h(z) e^{im\phi} \;\;\: m=0,\pm 1,..  $$

The radial wave function of the ground state can be expressed as

(6) $$ f(\rho)= \left\{ \begin{array}{ll} C_{w}\; J_{0} \left(\omega_{1} \rho\right) & \quad{\rho\leq \mathrm{R}}\\ C_{b}\; K_{0} \left(\omega_{2} \rho\right) & \quad{\rho >\mathrm{R} }\;\; \end{array} \right.  $$

where $\omega _{1}=\sqrt {\frac {2m^{*}_{w}}{\hbar ^{2}}E_{\bot }}$, $\omega _{2}=\sqrt {\frac {2m^{*}_{b}}{\hbar ^{2}}(V_{I}-E_{\bot })}$, *J*_0_ and *K*_0_ are the zero-order Bessel and modified Bessel functions. The wave function *h*(*z*) can be obtained by the linear combination of Airy functions *A*_*i*_ and *B*_*i*_,

(7) $${} h(z)= \left\{ \begin{array}{ll} A_{i}\left(\frac{\eta_{1} z-{\eta_{2}^{2}}}{\left(-\eta_{1}\right)^{2/3}}\right)+ B_{i} \left(\frac{\eta_{1} z-{\eta_{2}^{2}}}{\left(-\eta_{1}\right)^{2/3}} \right)& \quad \text{well region}\\ A_{i}\left(\frac{\kappa_{1} z+{\kappa_{2}^{2}}}{\left(-\kappa_{1}\right)^{2/3}}\right)+ B_{i}\left(\frac{\kappa_{1} z+{\kappa_{2}^{2}}}{\left(-\kappa_{1}\right)^{2/3}}\right) & \quad \text{barrier region }\;\; \end{array} \right.  $$

where $\kappa _{1}=\sqrt {\frac {2m^{*}_{b} eF}{\hbar ^{2}}}$, $\kappa _{2}=\sqrt {\frac {2m^{*}_{b}}{\hbar ^{2}}(V_{\textit {II}}-E_{||})}$, $\eta _{1}=\sqrt {\frac {2m^{*}_{w} eF}{\hbar ^{2}}}$, $\eta _{2}=\sqrt {\frac {2m^{*}_{w}}{\hbar ^{2}}E_{||}}$. Matching the wave functions and their derivatives at the boundaries, the normalization constants and energy eigenvalues, *E*_0_=*E*_||_+*E*_⊥_, are determined.

The wave function for $\hat {H}$ is obtained by variational method,

(8) $$ \Phi=\psi(\rho,\phi,z)\;e^{-\alpha\left(\rho-\rho_{i}\right)^{2}-\beta\left(z-z_{i}\right)^{2}}  $$

where *α* and *β* are the variational parameters and *ρ*_*i*_ and *z*_*i*_ are the position of the impurity.

*b) Magnetic field*

The Hamiltonian for the system in the presence of magnetic field is

(9) $$ \hat{H}_{0}=\frac{\left(\hat{P}-\frac{e}{c} \vec{A}\right)^{2}}{2m^{*}} + V(\rho,z)  $$

where $\vec {A}$ is the vector potential of magnetic field. For a homogeneous magnetic field $\vec {B}(0,0,B)$ directed along z-axis, the vector potential is chosen as $\vec {A}=\left (\vec {B}\times \vec {r}\right)/2$. The wave function for *H*_0_ can be written as Eq. (5), where the ground state radial wave functions *f*(*ρ*) are Whittaker functions and *h*(*z*) is the linear combination of sin(*γ* *z*) and cos(*γ* *z*) functions,

(10) $$ f(\rho)=\left\{ \begin{array}{ll} \frac{C^{\prime}_{w}}{\rho} WM\left(\frac{{\gamma_{1}^{2}}}{2\mu},0,\frac{\mu \rho^{2}}{2}\right) &\quad {\rho\leq \mathrm{R}}\\ \frac{C^{\prime}_{b}}{\rho} WW\left(\frac{-{\gamma_{2}^{2}}}{2\mu},0,\frac{\mu \rho^{2}}{2}\right) &\quad {\rho > \mathrm{R}}\;\; \end{array} \right.  $$

where $\gamma _{1}=\sqrt {E_{\bot }/R^{*}}$, $\gamma _{2}=\sqrt {(V_{I}-E_{\bot })/R^{*}}$, $R^{*}=m^{*}e^{4}/ (2c^{2}\hbar ^{2})$ and $\mu =\hbar e B_{0}/(2m^{*} c R^{*})$. The impurity binding energy, *E*_*b*_, is obtained as

(11) $$ E_{b} =E_{0} - \min_{\alpha, \beta} \frac{<\Phi|\hat{H}|\Phi>}{<\Phi|\Phi>}.  $$

## Results and discussion

In this study, the numerical calculations are carried out on a typical *G* *a* *N*/*I* *n*_*x*_ *G* *a*_1−*x*_ *N*/*I* *n*_*y*_ *G* *a*_1−*y*_ *N* QDs. The following parameters are used in the calculations: *m* ^∗^=[0.1*x*+0.19(1−*x*)]*m*_0_, *E*_*g*_=3.22(1−*x*)+1.9*x*−1.4*x*(1−*x*), *x*=0.15, and *y*=0.02.

**Figure 2:**
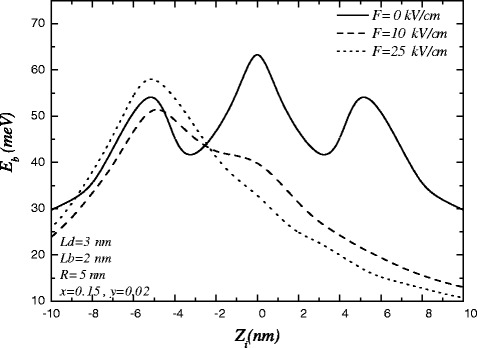
**The variations of binding energy in terms of impurity position in the presence of electric fields.**

Figure 2 is shown the impurity binding energy as a function of impurity position for different electric fields strength. For *F*=0 the binding energy has a maximum value when the impurity is located at the center of the inner dot. Also, the binding energy has two weaker symmetric local maxima for the impurity in the outer dots. The reason is that the electron wave function is mainly distributed inside the well region of the dots, and the Coulomb interaction is considerably large when the impurity is at the center of dots. The results are similar to works done by Wei, Chang and Zheng [16,19]. For *F*≠0, the electron is under electric force and its wave function is pushed to left side of the system. In this case the variation of binding energy is not symmetric, it has only a maximum value when the impurity is located at the center of QD1.

**Figure 3:**
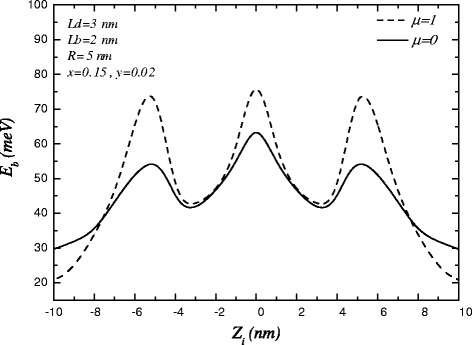
**The variations of binding energy in terms of impurity position in the presence of magnetic field.**

The variation of binding energy in terms of impurity position under magnetic field along z-direction is presented in Figure 3. As it is seen, the binding energy increases as the magnetic field is applied. Because of, the magnetic field causes the wave function of electron localizes inside the dot regions and, therefore, the interaction of electron and impurity located in the center of QDs increases.

**Figure 4:**
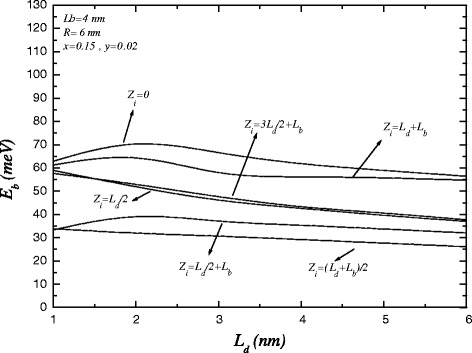
**The variations of binding energy versus the length of cylindrical quantum dot for various impurity positions.**

Figure 4 displays the binding energy as a function of the height of dot, *L*_*d*_, for different impurity positions. When the impurity is at the center of dots (*z*_*i*_=0,*z*_*i*_=*L*_*d*_+*L*_*b*_), the binding energy increases, it reaches a maximum for a given *l*_0_ and then decreases as the dot height increases. It is due to the electron wave function is localized inside the dot for *L*_*d*_≤*l*_0_≃2.3 nm, and then the Coulomb interaction is increased but for *L*_*d*_>*l*_0_ the distance between electron and impurity is increased and the binding energy decreases. For impurity position at the center of barrier (*z*_*i*_=(*L*_*d*_+*L*_*b*_)/2), the binding energy decreases as *L*_*d*_ increases, this is because the interaction between electron and impurity decreases. The binding energy for impurity position at the end of QDs (*z*_*i*_=*L*_*d*_/2, 3*L*_*d*_/2+*L*_*b*_) decreases as *L*_*d*_ increases. This is also because the wave function is distributed at the dot and the distance between electron and impurity increases as *L*_*d*_ increases.

**Figure 5:**
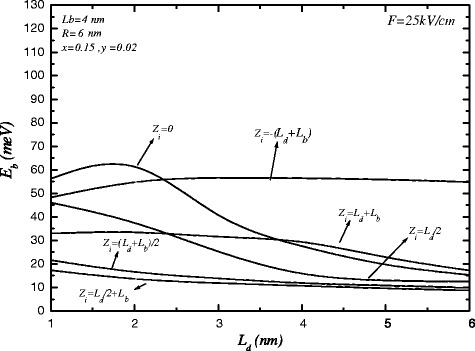
**The variations of binding energy versus the length of cylindrical quantum dot for various impurity positions in the presence of electric field.**

The effect of external electric field on the binding energy on symmetric multiple QDs is considered in Figure 5. As it is seen, the maximum value of binding energy for impurity at the center of dot decreases and is shift to left (*L*_*d*_<2 nm). The presence of electric field causes the wave function is pushed to the left direction and the binding energy for impurity positions at the left side of system, such as *z*_*i*_=−(*L*_*d*_+*L*_*b*_) increases, while it decreases for impurity positions at the right side.

**Figure 6:**
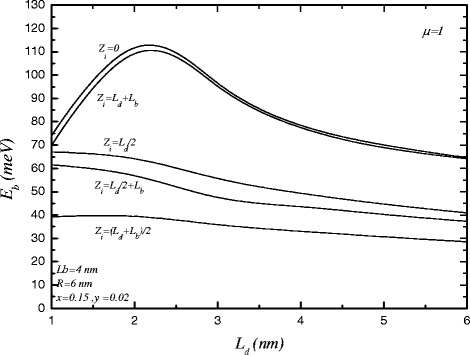
**The variations of binding energy versus the length of cylindrical quantum dot for various impurity positions in the presence of magnetic field.**

In Figure 6, the variation of the binding energy as a function of *L*_*d*_ and in the presence of magnetic field is shown. The binding energy increases for all impurity positions. For *z*_*i*_=0 and *z*_*i*_=*L*_*d*_+*L*_*b*_, the binding energies have the similar variations and almost the same value. The reason is the same as Figure 3.

## Conclusion

The binding energy in a multiple cylindrical quantum dots using the variational method and appropriate wave function is calculated for ZB *GaN* structures in the presence of electric and magnetic fields. The results clearly showed that the binding energy has three peaks, that are around the center of dots, and decreases as the dot size increases. The electric field varies the value and the position of binding energy peaks according to impurity positions. The binding energy increases as magnetic field is applied for all impurity positions. The behavior of the binding energy is dependent on distribution of electron wave function and impurity position.
